# Enterovirus A71 Proteins: Structure and Function

**DOI:** 10.3389/fmicb.2018.00286

**Published:** 2018-02-21

**Authors:** Jingjing Yuan, Li Shen, Jing Wu, Xinran Zou, Jiaqi Gu, Jianguo Chen, Lingxiang Mao

**Affiliations:** ^1^Department of Laboratory Medicine, The Affiliated People's Hospital, Jiangsu University, Zhenjiang, China; ^2^Clinical Laboratory, Danyang People's Hospital, Jiangsu, China; ^3^Clinical Laboratory, Zhenjiang Center for Disease Control and Prevention, Jiangsu, China; ^4^Institute of Laboratory Medicine, School of Medicine, Jiangsu University, Zhenjiang, China

**Keywords:** enterovirus-A71, protein, structure, function, epitope

## Abstract

Enterovirus A71 (EV-A71) infection has grown to become a serious threat to global public health. It is one of the major causes of hand, foot, and mouth disease (HFMD) in infants and young children. EV-A71 can also infect the central nervous system (CNS) and induce diverse neurological complications, such as brainstem encephalitis, aseptic meningitis, and acute flaccid paralysis, or even death. Viral proteins play a crucial role in EV-A71 infection. Many recent studies have discussed the structure and function of EV-A71 proteins, and the findings reported will definitely aid the development of vaccines and therapeutic approaches. This article reviews the progress in the research on the structure and function of EV-A71 proteins. Available literature can provide a basis for studying the pathogenesis of EV-A71 infection in detail.

## Introduction

EV-A71 is one of the major etiological agents causing hand, foot, and mouth disease (HFMD), which generally affects children aged five and below. In addition to HFMD, EV-A71 can cause various neurological complications, including aseptic meningitis, brainstem encephalitis, acute flaccid paralysis, neurogenic pulmonary edema, delayed neurodevelopment, and reduced cognitive function (Ooi et al., 2010). In fact, severe pediatric complications involving the central nervous system (CNS) can even cause death. EV-A71 was first isolated in 1969 from the stool samples of children with CNS-related complications, in California, USA (Schmidt et al., 1974). Since then, several outbreaks have been reported worldwide. For example, an outbreak of EV-A71 caused 29 deaths in Sarawak, Malaysia, in 1997 (Chan et al., 2000). Following that, a large outbreak in Taiwan involved over 100,000 cases, which led to 78 fatalities (Ho et al., 1999). HFMD caused by EV-A71 infection was prevalent in many provinces and cities of mainland China during 2008–2009; it caused the death of 479 children (Sun et al., 2011; Zhang et al., 2011). From 2009 to 2016, the incidence of HFMD caused by EV-A71 in mainland China was on the higher side (Tan et al., 2011). EV-A71 epidemic has resulted in many fatalities because of serious neurological complications, and has since been identified as a serious threat to public health.

**Figure 1:**
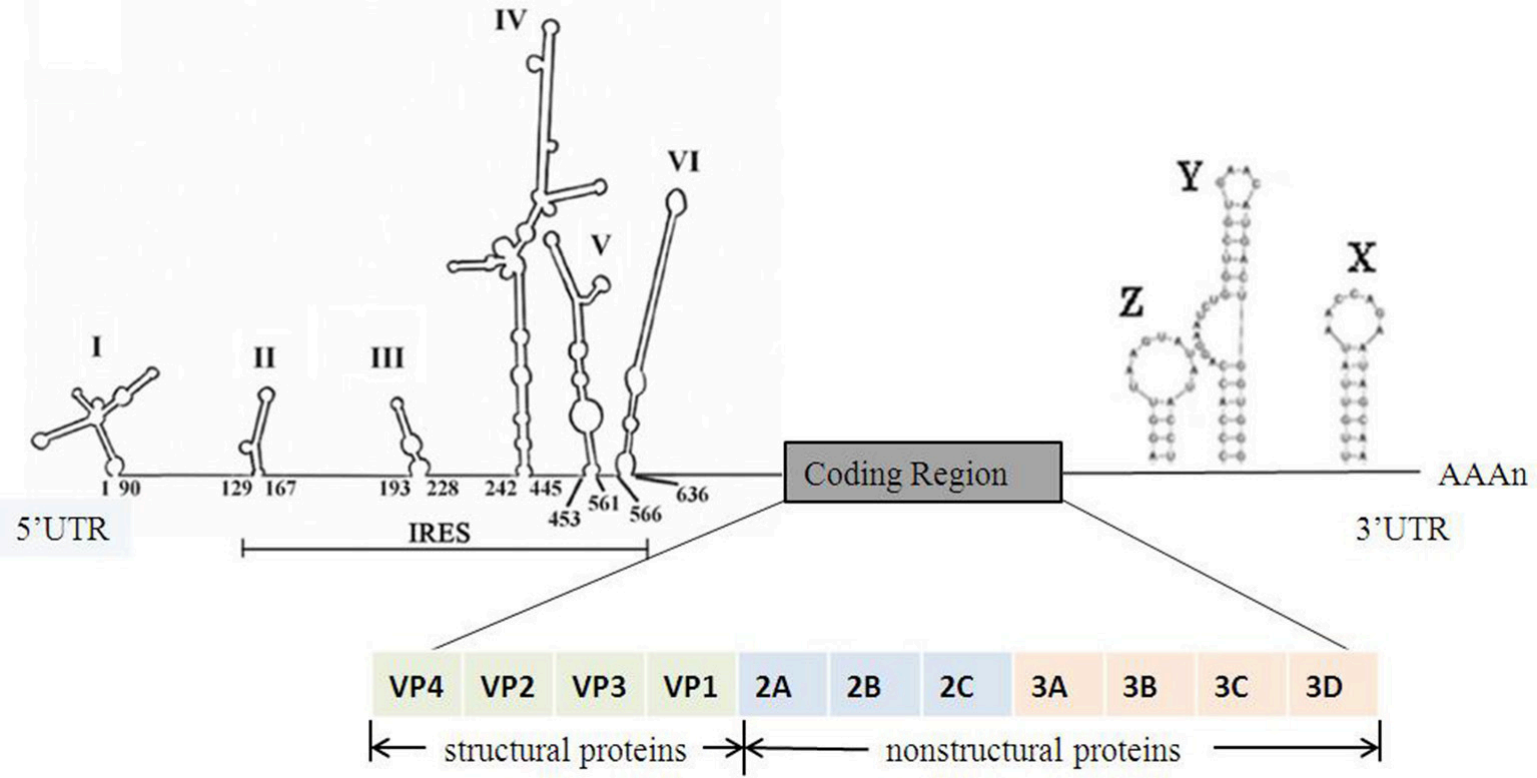
The structure of the EV-A71 genome. The 5′UTR of EV-A71 contains six stem-loop structures (I-VI) (Lin et al., 2009a). Stem loop I functions in negative strand synthesis, whereas stem loops II–VI form the type I IRES element. The ORF encodes a polyprotein which is cleaved into 11 viral proteins including 4 structural proteins and 7 non-structural proteins. The 3′UTR of EV-A71 contains three stem-loop structures (X, Y, and Z) which are associated with viral RNA replication (Kok et al., 2012).

**Figure 2:**
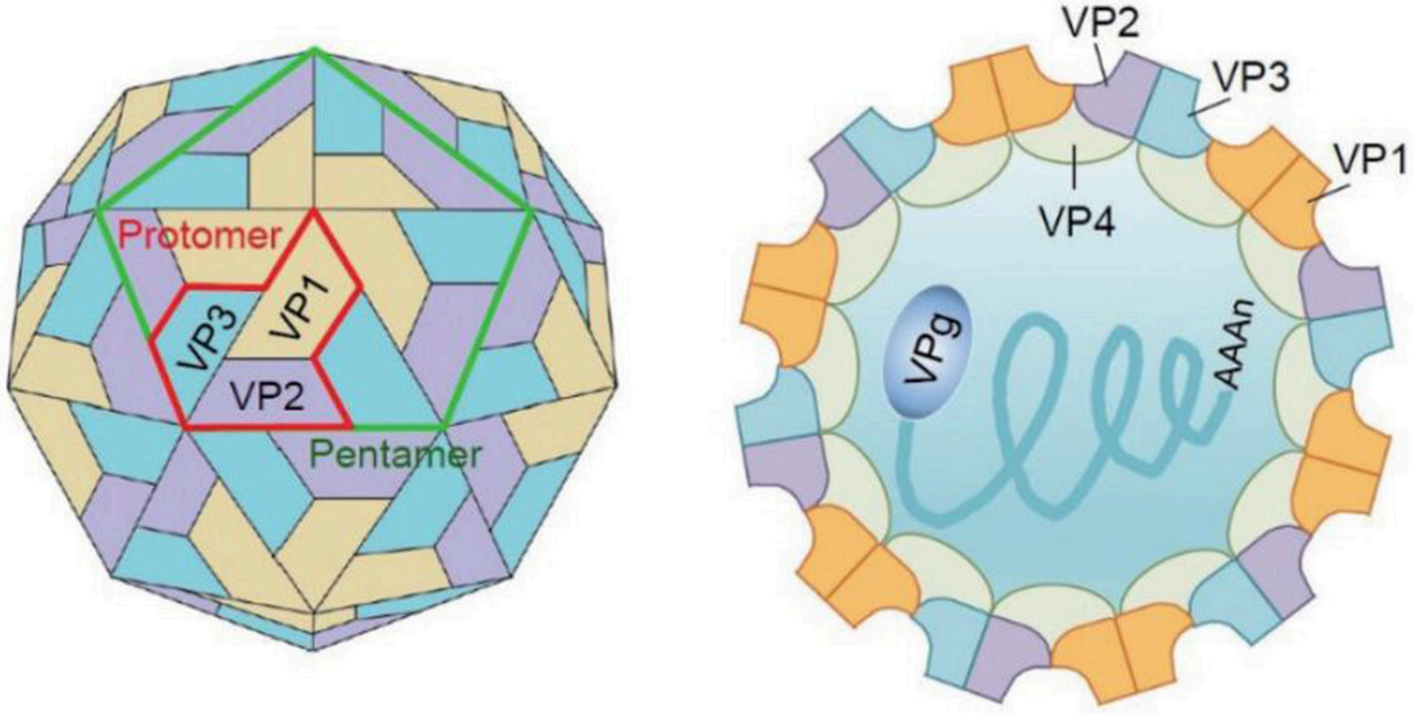
The structure of the virion. Three viral structural proteins (VP1–VP4) function as a single structural subunit, the protomer. Five protomers then form a pentamer, twelve of which can self-associate to form a capsid enclosing the viral genome. VP1, VP2, and VP3 are external of the virion, whereas VP4 is completely internalized in the virion (Yi et al., 2017).

EV-A71 belongs to the *Picornaviridae* family, genus *Enterovirus*, species Enterovirus A. The EV-A71 particle is non-enveloped, symmetrical, with a 20–30 nm icosahedral capsid. The viral genome is ~7,500-nucleotide-long, in the form of a single-stranded positive-sense RNA with only one open reading frame (ORF), flanked by a highly structured 5′-untranslated region (5′UTR) and a 3′UTR with a poly(A) tail. The structure of EV-A71 genome is shown in Figure 1. The ORF encodes a single large polyprotein of ~2,100 amino acids. The polyprotein is further hydrolyzed to form three precursor proteins, namely, P1, P2, and P3. P1 precursor protein is degraded into four

structural viral proteins, including VP1, VP2, VP3, and VP4. These four proteins assemble to form a protomer. Five such protomers constitute a pentamer, and 12 pentamers together form a virion enclosing the viral genome. VP1, VP2, and VP3 are exposed on the capsid surface, while VP4 is present inside the capsid. The structure of the virion is shown in Figure 2 (Yi et al., 2017). P2 and P3 encode seven non-structural proteins (P2–2A, 2B, 2C; P3–3A, 3B, 3C, 3D) (Solomon et al., 2010). The 5′UTR of EV-A71 contains six stem-loop structures (I–VI) (Thompson and Sarnow, 2003; Lin et al., 2009b). Stem-loop I is involved in viral RNA synthesis (Tu et al., 2007). Stem-loops II–VI comprise the internal ribosome entry site (IRES), which allows viral protein translation to occur in a cap-independent manner (Thompson and Sarnow, 2003). IRES can be positively regulated by the extracellular signal-regulated kinase 1/2 (ERK1/2), which is essential for the efficient replication of EV-A71 (Wang B. et al., 2012; Gao et al., 2014; Zhu et al., 2015). In addition, IRES binds to the cellular factor 68-kDa Src-associated protein during mitosis (Sam68), and the early growth response-1 (EGR1) facilitates EV-A71 replication (Song et al., 2015; Zhang et al., 2015). EV-A71 genome replication and RNA translation can be clearly inhibited when the cloverleaf structure and IRES are infected by sirtuin 1 (SIRT1) (Han et al., 2016). Thus, 5′UTR is essential for regulating EV-A71 replication and it is a potential drug target for treating EV-A71 infection. The 3′UTR of EV-A71 is a highly conserved domain, which contains three putative stem-loop structures (X, Y, and Z), followed by a poly(A) tail. Sequence analysis showed that X is the most conserved region, and Z, the least conserved, between the subgenogroups (Kok et al., 2012). Wang et al. proved that the point mutations introduced in 3′UTR to destabilize the kissing interaction, which is required for the maintenance of the overall structure of the enteroviral 3′UTR, resulted in a lethal phenotype. Therefore, the 3′UTR seems to play an important role in viral replication (Wang et al., 1999; Kok et al., 2012).

Individuals are infected with EV-A71 through the alimentary tract. The murine model showed that EV-A71 first replicates in the tonsils and lymph nodes, and subsequently goes through to the peripheral lymph node, leading to viremia. Then, the virus binds with specific receptors to initiate EV-A71 infection and intracellular replication (Chen et al., 2004). EV-A71 has several specific receptors: human P-selectin glycoprotein ligand 1 (PSGL-1), scavenger receptor B2 (SCARB2), human annexin II protein, sialylated glycan, and heparan sulfate. Compared to others, SCARB2 is considered the primary receptor of EV-A71 (Nishimura et al., 2009; Yamayoshi et al., 2009; Yang et al., 2009, 2011; Tan et al., 2013). EV-A71 enters host cells through specific receptors. At the same time, the polysaccharide and sialic acid on the cell surface play an important role in virus adsorption. Studies have found that the polysaccharide and sialic acid on the cell surface can be instrumental in allowing large number of viruses to enter the cells (Su et al., 2012). After binding to the viral receptors and entering the host cell via receptor-mediated endocytosis, the EV-A71 genome is translated in a cap-independent manner into a large polyprotein (Thompson and Sarnow, 2003), which is subsequently processed by the viral proteases 2A^pro^ and 3C^pro^ to form the structural capsid proteins (VP1–VP4) and the non-structural proteins (2A−2C, 3A−3D). The non-structural proteins are predominantly involved in the replication and translation of viral RNA (Bedard and Semler, 2004; Lin et al., 2009a). The parent RNA is used as the template for viral replication. During the replication of the whole virus, the genomic RNA directs the synthesis of the viral polyprotein, followed by the assembly and release of infectious virions. Studies on other picornaviruses have showed that translation and RNA replication cannot occur simultaneously on the same RNA molecule, indicating that there is a molecular switch to shut down RNA replication and allow initiation of translation (Wimmer et al., 1987; Shih et al., 2011). A comprehensive understanding of the structure and function of viral proteins will greatly help further the knowledge about the pathogenesis of EV-A71 as well as the strategies to resist EV-A71.

### Structural proteins

As mentioned earlier, the icosahedral capsid of EV-A71 consists of 60 identical protomers, each containing four different structural proteins (VP1–VP4). This viral capsid encloses single-stranded positive-sense RNA. During assembly, the P1 polyprotein is cleaved to VP0 (36 kDa), VP1 (32 kDa), and VP3 (27 kDa), and VP0 is subsequently cleaved into VP2 (28 kDa) and VP4 (8 kDa). VP1–VP3 are exposed on the capsid surface and span the thickness of the capsid, while VP4 is located inside the capsid (Plevka et al., 2012).

#### VP1 protein

VP1 is a capsid protein consisting of 297 amino acids. According to the crystal structure, VP1 is the most external part and the primary protein constituting two important structures: “canyon” and “pocket factor.” The depression around the icosahedral five-fold axes is called the “canyon” (Plevka et al., 2012). The hydrophobic “pocket factor” is mainly exposed on the surface of the canyon. The “pocket factor” can stabilize the virion. After EV-A71 infects the cell, VP1 binds with specific receptors (including PSGL-1 and SCARB2) (Nishimura et al., 2009; Yamayoshi et al., 2009; Yang et al., 2009, 2011; Tan et al., 2013), which have an immunoglobulin-like fold in the canyon. This immediately causes the expulsion of the hydrophobic “pocket factor” from the base of the “canyon,” leading to the destabilization of EV-A71. The destabilization is likely to be a prelude to genome release (Rossmann, 1994; Rossmann et al., 2002; Plevka et al., 2012). Receptor binding needs VP1-145 as a switch, which controls PSGL-1 binding by modulating the exposure of VP1-244K (Nishimura et al., 2013). Upon binding to the receptor(s), the EV-A71 virions undergo a two-step uncoating process, first conforming into an expanded, altered “A-particle,” which expels VP4 and exposes the N-terminus of VP1, subsequently anchoring the amphipathic helices into the membrane. Second, the EV-A71 A-particle capsid opens a two-fold channel near the icosahedral two-fold axis of symmetry to allow genome release, and forms a pore in the endosomal membrane to allow the genome to pass into the cytosol (Shingler et al., 2013). Additionally, the crystal structure of the EV-A71 uncoating intermediate showed that the N-terminal extensions of VP1 (residues 1–71) interact with the viral RNA (Lyu et al., 2014). Mutations in the VP1 Nter residues can increase cell tropism, which contributes to the pathogenesis of EV-A71 infection. The change in VP1 (244K→E) of a sub-genogroup B5 strain or VP1 (145Q→E) of a sub-genogroup C4 strain is the critical genetic determinant of mouse adaptation and virulence (Zaini and McMinn, 2012; Zaini et al., 2012). Moreover, VP1-145E variants are mainly responsible for the development of viremia and neuropathogenesis in a non-human primate model (Kataoka et al., 2015). In addition, VP1-activated endoplasmic reticulum (ER) stress and autophagy could promote the upregulation of Ecto-CRT (cell surface-exposed calreticulin), which is an important mediator for primary phagocytosis of viable neurons by the microglia (Hu et al., 2017).

VP1 has six surface loops (BC, DE, BE/aB, GF, GH, and HI), which are located around the icosahedral five-fold axes. The loops exposed on the virion surface are the most variable regions of EV-A71. The VP1 (97 L → R) substitution within the BC loop clearly increased neural cell tropism (Cordey et al., 2012). Ku et al. found that three neutralizing monoclonal antibodies bound the same conserved epitope located at the VP1 GH loop of EV-A71. Further, these monoclonal antibodies can inhibit both viral attachment and internalization during viral entry (Ku et al., 2015). Several studies showed that there is a neutralizing epitope close to the VP1 GH loop (Foo et al., 2007; Liu et al., 2011; Xu et al., 2015). In particular, this peptide (overlapping with the GH loop) lies on the capsid surface alongside the VP2 EF loop (residues 136–150) to form a functionally important epitope. Based on this, several monoclonal antibody (mAb) candidates with therapeutic potential were identified, such as mAb 2G8 (Deng et al., 2015), mAb51 (Lim et al., 2013), and mAb 22A12 (Shingler et al., 2015). The major epitopes in the structural proteins are shown in Table 1. Considering that EV-A71 VP1 is highly conserved and has many epitopes, most studies have focused on developing recombinant VP1 vaccines. Compared to inactivated vaccines, these are safer and more cost-effective. This showed the potential of recombinant VP1 vaccines as good vaccine candidates (Kiener et al., 2013; Yu et al., 2013; Xu and Zhang, 2016).

**Table 1:** Major epitopes in structural proteins.

**Region**	**Location**	**Name**	**Amino acid position**	**Peptide sequences**	**Characteristics of the epitope**	**References**
VP1	Neighboring location	PEP27	142–156	PTGEVVPQLLQYMFV	EV-A71-specific IgM epitope	Aw-Yong et al., 2016
		SP2	145–159	EVVPQLLQYMFVPPG	CD4+ T-cell epitopes	Foo et al., 2008
		VP1-20	145–162	EVVPQLLQYMFVPPGAPK	CD4+ T-cell epitopes	Tan et al., 2013
	Neighboring location	PEP23	41–55	TGEVPALQAAEIGAS	IgG epitope	Aw-Yong et al., 2016
		VP1-15	43–54	KVPALQAAEIGA	IgG epitope	Gao et al., 2012
		vp1-14	40–51	DTGKVPALQAAE	Anti-EV71 IgM epitope	Gao et al., 2012
	Neighboring location (close to the VP1 GH loop)	VP1-43	211–220	FGEHKQEKDL	Neutralization epitope	Liu et al., 2011
		SP70	208–222	YPTFGEHKQEKDLEYC	Neutralizing linear epitope	Foo et al., 2007
		VP1(aa208–222)	208–222	YPTFGEHKQEKDLEYC	Neutralizing linear epitope	Xu et al., 2015
VP2	Neighboring location (close to VP2 EF loop)	VP2-28	136–150	AGGTGTEDSHPPYKQ	Neutralization epitope	Liu et al., 2011
		VP2(aa141-155s) 7C7	141–155 142–146	TEDSHPPYKQTQPGA ED/NSHP	Neutralization epitope A linear, non-neutralizing epitope	Kiener et al., 2012; Xu et al., 2014
		PEP10	134–148	TVAGGTGTEDSHPPY	Neutralization epitope	Aw-Yong et al., 2016
		VP2–24	176–193	TVCPHQWINLRTNNCATI	T cell epitope	Tan et al., 2013
		A3	248–263	PHQWINLRTNNCATII	CD4+ T Cell Epitop	Wei et al., 2012
VP4		VP4N20	first 20 amino acids	GSGVSTGASGSHGASASATG	Neutralization epitope	Zhao et al., 2013

**Figure 3:**
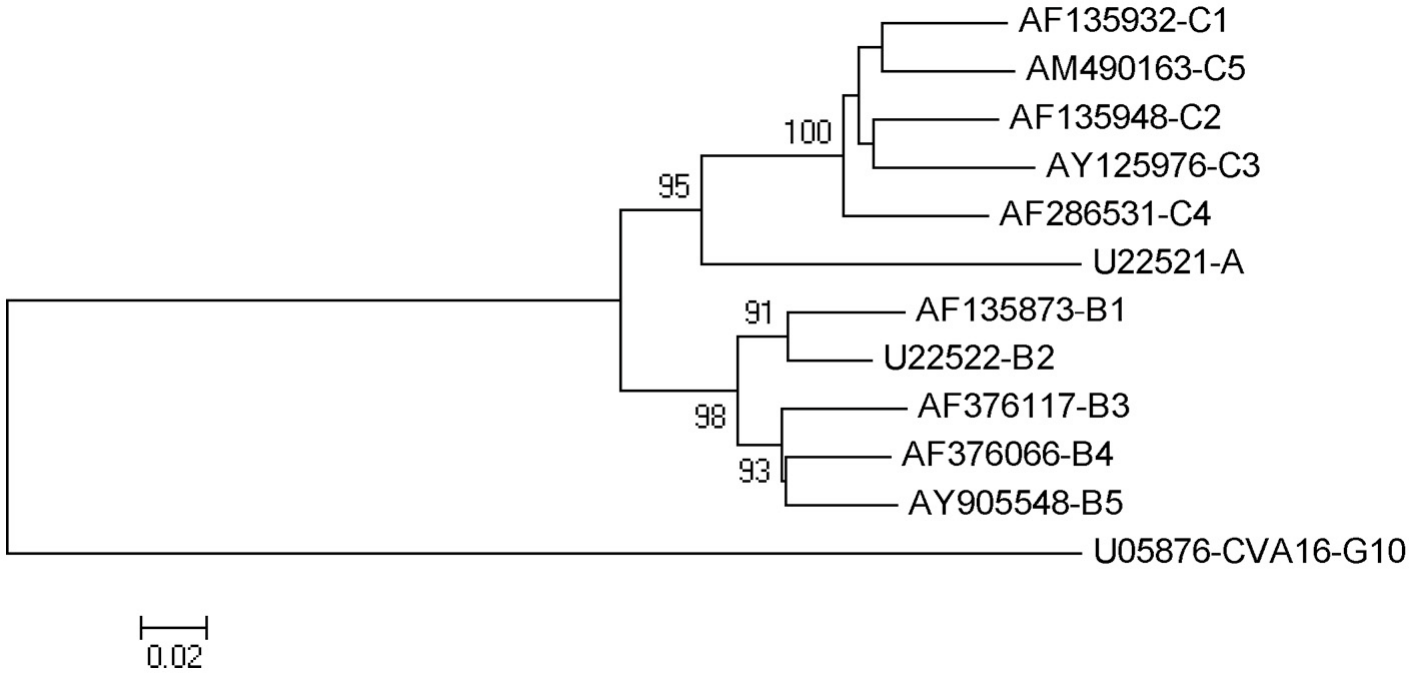
Phylogenetic tree constructed among EV71 strains of all genotype/ subgenotype based on complete sequence of vp1 coding gene. Note:bootstrap value (percentage of 1,000 pseudoreplicate datasets) supporting each cluster are shown at the node. Phylogenetic and molecular evolutionary analyses were conducted using MEGA version 5.0.

In addition, the VP1 gene is serotype-specific and considered as the most suitable region for sequence analysis (Wu J. S. et al., 2013; Wu W. H. et al., 2013; Duy et al., 2017). EV-A71 can be divided into three distinct genogroups (A, B, and C) (Brown et al., 1999). Genogroups B and C can be further divided into genogroups B1–B5 and C1–C5 (Solomon et al., 2010). A phylogenetic tree constructed for EV-A71 strains of all genotypes/subgenotypes based on the complete sequence of the VP1-coding gene is shown in Figure 3.

#### VP2-VP3 protein

VP2, VP3, and VP1 have the same topology: they form an eight-stranded anti-parallel β-barrel structure in the form of a wedge that facilitates packing. The form looks like β-sandwich “jelly-roll” folds. The main structural differences are the connecting loops and the C-termini on the outer side of the capsid (Kiener et al., 2012). In VP2, the most prominent surface loop is the “puff,” and in VP3, the largest protrusion on the surface is the “knob” (Plevka et al., 2012).

VP2 consists of 254 amino acids. VP2 has several neutralization epitopes, just as VP1 (Table 1). The epitope-mapping experiment showed a highly conserved linear epitope, namely, the residues 136–150 of VP2, which are highly conserved among the EV-A71 genotypes. VP2_136−150_ is not affected by formalin treatment and long-term storage, which makes it a surrogate biomarker in the potency testing of candidate EV-A71 vaccines (Liu et al., 2011). Xu et al. reported that there is a cross-neutralizing linear epitope, spanning amino acids 141–155, which lies in the large and highly variable surface loop of VP2 (Xu et al., 2014). Kiener et al. also showed a single, linear, non-neutralizing epitope, spanning amino acids 142–146, located in the EF loop of EV-A71 VP2. The S/T (144) mutation in this epitope confers loss of VP2 antigenicity to some new EV-A71-C4 strains from China (Kiener et al., 2012). A mutant virus with lysine to methionine substitution at VP2_149_ (VP2-149M) or glutamine to glutamic acid substitution at VP1-145 (VP1-145E) showed higher viral titers and increased apoptosis. The synergistic effect of VP2-149M and VP1-145E double mutations enhanced viral binding and accumulation of EV-A71 RNA, contributing to viral infectivity *in vitro* and mouse lethality *in vivo* (Huang et al., 2012).

VP3 consists of 245 amino acids, among which the amino acids 59–67 of VP3 are more highly conserved between the subgenogroups, compared to VP1. There is a conserved conformational epitope on the “Knob” region of VP3, which makes it an ideal target for a diagnostic or therapeutic mAb. The mAb 10D3 can generally recognize this conservative epitope, not coxsackievirus A16 (CVA16), which makes mAb 10D3 a valuable tool for differential diagnosis (Kiener et al., 2014). Recently, a therapeutic IgG antibody, 5H7, was confirmed to recognize a conformational epitope, which was mapped to the highly conserved amino acid position 74 of VP3 (Jia et al., 2017).

#### VP4 protein

VP4 consists of 69 amino acids. VP4 has an extended conformation and is present inside the virion. It is myristylated and confers stability to the capsid (Chow et al., 1987). VP4 gene is more conserved than VP1, VP2, and VP3 genes, and therefore, some studies on the EV-A71 vaccine focused on whether the VP4 protein contains neutralizing epitopes. The study further identified a highly conserved linear neutralizing epitope in the N-terminus of EV-A71 VP4 by epitope-mapping experiments. In the present study, the peptide containing N-terminal residues 1–20 of EV-A71 VP4 of the genotype C4 was fused to hepatitis B core antigen (HBcAg) and expressed in *Escherichia coli*. This fusion protein was able to spontaneously assemble into chimeric virus-like particles (VLPs), which elicited a virus-neutralizing antibody response. The results suggest that chimeric HBcAg particles carrying a neutralizing epitope of EV-A71 VP4 can be a promising vaccine candidate against EV-A71 infection (Zhao et al., 2013). As mentioned earlier, during viral uncoating, after the formation of the “A-particle,” the VP4 proteins are expelled and inserted into the membrane to form a channel through which the RNA can enter the host cell cytoplasm (Shingler et al., 2013). VP4 gene was also thought to be suitable for sequence analysis of EV-A71, just like VP1 (Chu et al., 2001; Ishiko et al., 2002; Shimizu et al., 2004).

The primary functions of EV-A71 structural proteins are shown in Table 2.

**Table 2:** The functions of EV-A71 structural proteins.

**Protein**	**Functions**	**References**
VP1	Receptor binding epitopes	Nishimura et al., 2009; Yamayoshi et al., 2009; Yang et al., 2009, 2011; Tan et al., 2013
	Increase cell tropism	Cordey et al., 2012; Zaini et al., 2012; Kataoka et al., 2015
	Regulate EV-A71 maturation	Zhang et al., 2017
	Genotyping	Wu C. et al., 2013; Wu J. S. et al., 2013; Duy et al., 2017
VP2	Potency test biomarker of candidate EV-A71 vaccines	Liu et al., 2011
	Epitopes	
VP3	mAb target	Kiener et al., 2014
	Therapeutic antibody target	Jia et al., 2017
VP4	Epitopes	

### Non-structural proteins

#### 2A protein

EV-A71 2A proteinase (2A^pro^) is a cysteine proteinase and contains ~150 amino acids. According to the crystal structure, 2A^pro^ maintains a chymotrypsin-like fold. The active site is composed of the catalytic triads, namely, C110A, H21, and D39. Moreover, 2A^pro^ contains a tightly folded cI-to-eI2 loop at the N-terminal domain and renders a hydrophilic surface for host protein recognition. At the C-terminal of 2A^pro^, there is an important structure composed of a hydrophobic motif “LLWL,” followed by an acidic motif “DEE.” The “LLWL” motif is folded into a previously unknown double β-turn structure, which is essential for the positioning of the acidic motif. The acidic motif and the preceding “LLWL” motif at the C-terminus of 2A^pro^ are essential for viral replication (Mu et al., 2013).

The protease activity of 2A^pro^ deems 2A^pro^ important in viral replication, apoptosis, pathogenesis, and other processes. First, 2A^pro^ is responsible for shearing the synthesized poly-proteins. 2A^pro^ theoretically catalyzes the peptide bond cleavage between VP1 (C-terminal) and P2 (N-terminal of 2A protein). 2A^pro^ can also cleave 3CD to produce 3C′ and 3D′. In addition, 2A^pro^ exhibited strong transcriptional activity in yeast cells, which was independent of its protease activity. The transcriptional activity of EV-A71 2A^pro^ plays a role in viral replication and/or pathogenesis (Yang et al., 2010). Li et al. demonstrated that 2A^pro^ modulates the replication and virulence of EV-A71 by testing the virulence of 2A^pro^-replaced chimeric strains (Li C. et al., 2017). As mentioned earlier, ERK is crucially involved in the positive regulation of EV-A71 IRES. Duan et al. demonstrated that the positive regulation of viral replication by the ERK cascade was mediated by affecting both cis-cleavage of the viral polyprotein by 2A^pro^ and trans-cleavage of the cellular eIF4GI (Duan et al., 2017). Additionally, Wu et al. observed that the expression of EV-A71 2A^pro^ alone was sufficient to cause stress granule formation (Wu et al., 2014). 2A^pro^ cleaves the host translation initiation factor, the eukaryotic translation initiation factor 4-gamma 1(eIF4G1), to induce apoptosis (Kuo et al., 2002).

2A^pro^ uses some mechanisms to escape innate immunity. 2A^pro^ and 3C^pro^ cleave NLRP3 (NOD-like receptor family, pyrin domain containing 3) protein, which plays a protective role against EV-A71 infection, leading to EV-A71 counteracting inflammasome activation (Wang H. et al., 2015). 2A^pro^ contributes to the evasion of innate immunity by cleaving Type I interferon (IFN)-α/β receptor 1 (IFNAR1) to block IFN-induced Jak/STAT signaling (Liu et al., 2005; Yi et al., 2011). This makes 2A^pro^ a potential drug target for anti-EV-A71-infection activities. CW-33 combined with a low-dose of Type I IFN can be used to treat EV-A71 infection by inhibiting 2A^pro^ activity (Wang C.Y. et al., 2015). 2A^pro^ is able to cleave the nuclear pore glycoprotein protein 62 (Nup62), disrupting host nuclear transport pathways and altering nuclear permeability (Zhang et al., 2013). 2A^pro^ can specifically slice mitochondrial antiviral signaling (MAVS) protein, inactivating the antiviral innate immune response of retinoic acid-induced gene-I (RIG-I) and melanoma differentiation-associated gene, thereby lowering the production of Type I IFN (Wang et al., 2013). In addition, Wang et al. showed that 2A^pro^ attenuated IFN-γ signaling by reducing serine phosphorylation of signal transducers and activators of transcription 1 (STAT1), after the inactivation of extracellular signal-regulated kinases without affecting STAT1 expression (Wang L. C. et al., 2015).

#### 2B protein

EV-A71 2B protein is an ion channel protein and contains ~100 amino acids. It has two transmembrane domains(TM1 and TM2). Xie et al. demonstrate that EV71 2B protein forms an ion channel and chloride is the principal ion carried in the 2B-mediated current. As a cytolytic virus, EV71 may primarily release its progeny via cell lysis. The 2B-mediated, chloride- dependent current may perturb anion homeostasis in the Golgi complex and ultimately increase virus production. 4,4′-diisothiocyano-2,2′-stilbenedisulfonic acid (DIDS) can suppress virus release by inhibiting the 2B protein-mediated chloridedependent current (Xie et al., 2011).

Cong et al. reported that 2B induces cell apoptosis and affects the mitochondrial apoptotic pathway by directly modulating the redistribution and activation of the proapoptotic protein Bax. After EV-A71 infection, 2B protein is localized to the mitochondria. Then, it interacts with and activates Bax to induce cell apoptosis (Cong et al., 2016).

#### 2C protein

EV-A71 2C protein mainly functions as an NTPase and contains 329 amino acids. It consists of three subdomains: an ATPase domain, a zinc finger, and a long protruding C-terminal α-helix (PDB References: 5GRB). Zinc fingers show trigonal bipyramidal geometry, with the zinc-binding site presenting a key difference among picornaviral 2C homologs. The long protruding C-terminal α-helix is proved to be involved in the self-oligomerization of 2C in solution (Guan et al., 2017).

The amino acids located at 5–43 on the N-terminus of 2C have potential membrane-binding activity. These amino acids are involved in the rearrangement of host cell membrane proteins and formation of viral replication complex. In this process, 2C mainly associates with the ER protein 3 of host cells and combines with the viral double-stranded RNA, thus forming a viral replication complex (Tang et al., 2007). Also, 2C can recruit COPI to the viral replication complex to ensure effective replication of EV-A71 (Wang J. et al., 2012).

2C is involved not only in viral replication, but also in innate immune evasion. Du et al. demonstrated that EV-A71 2C can reduce the formation of the predominant form of NF-κB (heterodimer p65/p50) by interacting with the IPT domain of RelA(p65) (Du et al., 2015). In addition, EV-A71 2C can inhibit IKKβ activation, thus blocking NF-κB activation (Zheng et al., 2011). These might be novel mechanisms adopted by EV-A71 to antagonize innate immunity.

#### 3A protein

EV-A71 3A protein is a membrane-bound protein, containing 86 amino acids. The N-terminus of 3A is rich in proline residues and is involved in protein–protein interactions. Like other enteroviruses, EV-A71 relies on phosphatidylinositol-4-kinase IIIβ (PI4KB) for genome RNA replication. 3A interacts with a host factor acyl-coenzyme A-binding domain-containing 3 (ACBD3) to recruit PI4KB to the genome replication sites, which facilitates viral RNA replication (Xiao et al., 2017). Lei et al. also showed that the Golgi resident protein ACBD3 can facilitate the replication of EV-A71 by interacting with 3A. The viral and host proteins form a large complex that is necessary for RNA synthesis at replication sites (Lei et al., 2017a). A recent study showed that EV-A71 3A can interact with human β3 subunit of Na+/K+-ATPase (ATP1B3) protein, which inhibit EV-A71 replication by enhancing the production of type-I IFN. Although the role of 3A in this response is not clear, this might be a mechanism by which 3A escapes innate immunity (Lu et al., 2016). It is worth mentioning that an FDA-approved drug, itraconazole (ITZ), has been identified as an effective inhibitor of EV-A71 replication, in the low-micromolar range, by targeting 3A (Gao et al., 2015).

#### 3B protein

EV-A71 3B protein, also known as VPg (virus genome-linked protein), is a nucleic acid chaperone protein and contains ~22 amino acids. The crystal structure of the EV-A71 3D^pol^-VPg complex showed that VPg was anchored at the bottom of the palm domain of the 3D^pol^ molecule and exhibited an extended V-shape conformation (Chen et al., 2013).

VPg uridylylation is essential for picornavirus RNA replication. In the context of viral genomic RNA, the mutations that abolished VPg uridylylation activity were lethal for EV-A71 replication (Chen et al., 2013). Sun et al. reported that Site-311, which located at the base of the palm domain of EV-A71 3D^pol^, is a VPg-binding site that stabilizes the VPg molecule during VPg uridylylation. Their research also suggested a two-molecule model for 3D^pol^ during EV-A71 VPg uridylylation, such that one 3D^pol^ presents the hydroxyl group of the third amino acid Tyr of VPg to the polymerase active site of another 3D^pol^, which, in turn, catalyzes VPg→VPg-pU conversion. These results indicate that the VPg uridylylation reaction involves the binding of VPg to 3D^pol^ and the transfer of uridine monophosphate (UMP) by 3D^pol^ to the hydroxyl group of the third amino acid Tyr of VPg (Sun et al., 2012).

#### 3C protein

EV-A71 3C protease (3C^pro^) is a cysteine protease containing 184 amino acids. Like 2A^pro^, 3C^pro^ has a typical chymotrypsin-like fold. Cui et al. found an important surface loop, denoted as the β-ribbon, which adopts a novel open conformation in EV-A71 3C^pro^. Two important residues, namely, Gly123 and His133, located at the base of the β-ribbon form hinges, and the hinge residues are important for the proteolytic activity of EV-A71 3C^pro^ (Cui et al., 2011). However, Wu et al. reported (PDB References: 4ghq) that the conformation of β-ribbon was the same as that in all other picornavirus 3C^pro^ structures, which constituted an essential part of the substrate-binding cleft, but differed from the one discussed in a previous article but differs from a previous structure in a different space group (Wu C. et al., 2013).

EV-A71 3C^pro^ plays an irreplaceable role in segmenting the precursor polyprotein during viral replication. During viral replication, 3C^pro^ cuts itself from the P3 precursor protein, and then cuts P2 and P3 to form viral proteins. Moreover, 3C^pro^ was found to possess RNA-binding activity. Mutations in EV-A71 3C^pro^ influenced RNA-binding activity and proteolytic activity (Shih et al., 2004). Recently, 3C^pro^ has been found to contain a novel virulence determinant involved in EV-A71 infection. The recombinant virus with a single point variation in the 69th residue of 3C^pro^ exhibited an obvious decline in replication and virulence. Thus, the 69th residue of 3C^pro^ has been identified as a novel virulence determinant of EV-A71 (Li B. et al., 2017). Ma et al. recently confirmed that the polymorphisms of EV-A71 3C^pro^ at the 79th amino acid position were associated with clinical severity and viral replication, which might be related to the interaction of 3C^pro^ with important host proteins such as tripartite motif-containing protein 21 (TRIM21) (Ma et al., 2017). EV-A71 3C^pro^ could interfere with the polyadenylation of host mRNA by digesting the host protein CstF-64, a cleavage-stimulating factor responsible for 3′pre-mRNA cleavage and polyadenylation (Weng et al., 2009).

EV-A71 3C^pro^ plays a critical role in the immune evasion of EV-A71 infection. Transforming growth factor-β-activated kinase 1 (TAK1) plays a role in the formation of TAK1/TAB1 (TAK1-binding protein1)/TAB2/TAB3 complex. TAK1, TAB1, TAB2, and TAB3 are all essential for downstream NF-κB activation. Lei et al. showed that EV-A71 3C^pro^ suppresses cytokine expression via cleavage of the TAK1/TAB1/TAB2/TAB3 complex. On the other hand, EV-A71 disables the components of TAB2 complex by 3C^pro^-mediated cleavage of TAB2 and its partners, while the overexpression of TAB2 inhibits EV-A71 replication (Lei et al., 2014). Further, EV-A71 3C^pro^ can inhibit host immune response by cleaving the adaptor protein TRIF (TIR-domain-containing adapter-inducing interferon-β) and IFN regulatory factor 7 (IRF7) (Lei et al., 2010, 2013).

In addition, 3C^pro^ can regulat cell pyroptosis and apoptosis through different pathways. Lei et al. demonstrated that EV-A71 3Cpro directly cleaves gasdermin D (GSDMD), an important component of pyroptosis, which can regulate lipopolysaccharide and NLRP3-mediated secretion of interleukin-1β (IL-1β), resulting in inhibiting cell pyroptosis (Lei et al., 2017b). Li et al. used the human glioblastoma SF268 cell line to investigate the induction of apoptosis by EV-A71 3C^pro^. They found that the proteolytic activity of 3C^pro^ triggers apoptosis in the SF268 cells through a mechanism involving caspase activation, and that this apoptotic pathway might play an important role in the pathogenesis of EV-A71 infection (Li et al., 2002). 3C^pro^ also promoted apoptosis by cleaving PinX1 (a telomere-binding protein) using its protease activity, and that this cleavage facilitated EV-A71 release (Li J. et al., 2017). Further, it was reported that SUMOylation (where SUMO is small ubiquitin-like modifier) promotes EV-A71 3C^pro^ ubiquitination, leading to degradation, correlating with a decrease in viral replication and cell apoptosis. 3C^pro^ can be SUMO-modified at the residue Lys 52 by the SUMO E2-conjugating enzyme Ubc9. SUMOylation down-regulated 3C^pro^ activity *in vitro* and 3C^pro^ stability in the cells (Chen et al., 2011). Because 3C^pro^ is irreplaceable in viral replication, some antiviral drugs are targeted at 3C^pro^, such as DC07090 (Ma et al., 2016) and luteoloside (Cao et al., 2016).

#### 3D protein

EV-A71 3D polymerase (3D^pol^), containing ~462 amino acid, works as an RNA-dependent RNA polymerase (RdRp). The overall crystal structure of EV-A71 3D^pol^ (PDB References: 3N6L) adopts the usual closed “right-hand” conformation observed in other RdRps, which is composed of “fingers,” “palm” and “thumb” domains. 3D^pol^ has six conserved motifs, each containing four-amino-acid-long sequences, namely, motif A, B, C, D, E, and F, respectively. Motifs A, B, C, and D are located in the palm domain, whereas motif E is in the palm domain, and motif F, in the fingers domain. The crystal structure also showed that 3D^pol^ (RdRp) is a specific Mn^2+^-dependent polymerase (Wu et al., 2010).

3D^pol^ is responsible for RNA replication, just like RdRp. Additionally, 3D^pol^ is involved in VPg uridylation (Sun et al., 2012). The site-311 located at the base of the palm domain of 3D is a VPg-binding site, which stabilizes the VPg molecule. Liu et al. demonstrated that 3D^pol^ is modified by SUMO-1, both during infection and *in vitro*. More importantly, increasing the level of SUMO-1 in EV-A71-infected cells augmented the SUMOylation and ubiquitination levels of 3D^pol^, leading to enhanced replication of EV-A71 (Liu et al., 2016). Yu et al. found that 3D^pol^ induces the modulation of the expression profile of S-phase control proteins, a function that is consistent with its role in arresting S phase. The study suggested that 3D^pol^ is primarily responsible for S phase arrest caused by EV-A71 (Yu et al., 2015). Moreover, EV-A71 3D^pol^ was found to stimulate the activation of NLRP3 inflammasome and the release of IL-1β through direct binding to NLRP3. 3D interacts with NLRP3 to facilitate the assembly of inflammasome complex by forming a 3D-NLRP3-ASC (the apoptosis-associated speck-like protein possessing a caspase-recruiting domain) ring-like structure, resulting in the activation of IL-1β. These findings demonstrate that 3D^pol^ plays an important role in the activation of inflammatory response (Wang et al., 2017). 3D^pol^ may also contribute to evasion of innate immunity. 3D^pol^ attenuated IFN-γ signaling accompanied by a decrease in STAT1 expression, without interfering with the expression of IFN-γ receptor (Wang L. C. et al., 2015). Some medicines targeting 3D^pol^, such as baicalin (Li et al., 2015) and aurintricarboxylic acid (Hung et al., 2010), exhibit potent antiviral effect.

The primary functions and related pathways of EV-A71 non-structural proteins are shown in Table 3.

**Table 3:** The functions and pathways of EV-A71 non-structural proteins.

**Protein**	**Functions**	**Pathway**	**References**
2A	Facilitate virus replication	Cleave the poly-protein; transcriptional activity; Mediate ERK signaling	Yang et al., 2010; Duan et al., 2017; Li B. et al., 2017
	Induce cell apoptosis	eIF4G1	Kuo et al., 2002
	Inhibit nuclear transport	Nup62	Zhang et al., 2013
	Immune evasion	NLRP3	Wang C.Y. et al., 2015
		IFNAR1(Jak/STAT signaling)	Liu et al., 2005; Yi et al., 2011
		MAVS, RIG-1, IFN-I	Wang et al., 2013
		The serine phosphorylation of STAT1	Wang H. et al., 2015
2B	Increase virus production	Chloride-dependent current	Xie et al., 2011
	Induce cell apoptosis	Mitochondrial apoptotic pathway	Cong et al., 2016
2C	Facilitate virus replication	Endoplasmic reticulum protein 3,COPI	Tang et al., 2007; Wang B. et al., 2012
	Immune evasion	p65	Du et al., 2015
		IKKβ	Zheng et al., 2011
3A	Facilitate virus replication	ACBD3; PI4KB	Li B. et al., 2017; Xiao et al., 2017
	Immune evasion	ATP1B3	Lu et al., 2016
3B	Facilitate virus replication	VPg	Chen et al., 2013
3C	Facilitate virus replication	RNA-binding activity and proteolytic activity	Shih et al., 2004
		TRIM21	Ma et al., 2017
		CstF-64	Weng et al., 2009
	Immune evasion	TAK1/ TAB1 / TAB2/ TAB3	Lei et al., 2014
		TRIF,IRF7	Lei et al., 2010, 2013
	Inhibit pyroptosis	GSDMD	Lei et al., 2017b
	Induce apoptosis	PinX1	Li C. et al., 2017
		Caspase activation	Li et al., 2002
3D	Facilitate virus replication	VPg uridylation	Sun et al., 2012
	Induce S-phase arrest	Cyclin E1, CDK2 T160	Yu et al., 2015
	Active inflammatory response	NLRP3,IL-1β	Wang et al., 2017
	Immune evasion	STAT1,IFN-e	Wang H. et al., 2015

## Conclusion

EV-A71 has four different structural proteins and seven non-structural proteins. VP1–VP4 forms the icosahedral capsid of EV-A71. They are mainly involved in receptor binding, and contain neutralizing epitopes. Some vaccines targeting VP1–VP4 have been investigated. The seven non-structural proteins play different roles in processes such as viral replication, viral pathogenesis, and immune evasion. The primary functions of EV71 proteins are listed in Table 1. Although some functions of EV-A71 proteins have been discovered, thorough knowledge about these proteins is still lacking. Given that EV-A71 infection has emerged as a significant global concern, affecting the Asia-Pacific region in particular, development of an effective vaccine and therapeutic approach might be the best way to control this infection. Further detailed study of EV-A71 proteins will ultimately be useful for developing new vaccines and therapeutic agents.

## Author contributions

JY, LS, JW, XZ, JG, JC conceived of the work and discussed the content. YJ drafted the manuscript, and then LS, JW, XZ, JG, JC were responsible for revising it. LM critically reviewed, edited, and finalized the manuscript for submission.

### Conflict of interest statement

The authors declare that the research was conducted in the absence of any commercial or financial relationships that could be construed as a potential conflict of interest.
